# Editorial: Plant probiotics: recent and future prospects

**DOI:** 10.3389/fpls.2023.1254184

**Published:** 2023-08-07

**Authors:** Usha Chakraborty, Pramod Ramteke

**Affiliations:** ^1^ Department of Botany, University of North Bengal, Siliguri, West Bengal, India; ^2^ Department of Molecular Biology & Genetic Engineering, RTM Nagpur University, Nagpur, Maharashtra, India

**Keywords:** probiotics, endophytes, biostimulants, sustainable agriculture, PGPR, biostimulants, sustainable agriculture

Plant probiotics may directly influence the growth of plants through enhanced nutrition, especially solubilizing phosphates and other insoluble nutrients in soil that secrete hormones; they may also act indirectly through antagonistic mechanisms whereby pathogens are directly inhibited or induce defense mechanisms in the host. Plant growth promoting microorganisms (PGPMs) are classic examples of plant probiotics. There is no doubt that such microorganisms can provide the most sustainable and eco-friendly means of helping plants overcome various stresses, which in turn help in promoting their health, and hence, their productivity. The present Research Topic contains six research papers and two reviews that provide information on various aspects of plant probiotics.

Saini et al. in their research paper have highlighted how two contrasting daizotrophs, or PGPB, a symbiotic nodulating *Bradyrhizobium* and the non-nodulating endophytic bacterium *Gluconoacetobacter* function as plant growth promoters in soybean and rice. Based on both biochemical and molecular studies, during the interaction of both microorganisms with the two plants, it was shown that among the sets of interactions, rice–*Gluconoacetobacter* and soybean–*Bradyrhizobium* are more compatible than *vice versa*. The authors argued that rice initially expressed transcripts linked to hypersensitivity together with PR proteins because it was unable to recognize *Bradyrhizobium* as a beneficial microorganism and likely thought of it as a disease-causing microorganism. The ability of the glyphosate-tolerant *Pseudomonas resinovorans* SZMC 25872 strain to inhibit *Agrobacterium tumefaciens*, a plant pathogenic bacterium, was described in the article by Zhumakayev et al. Based on their detailed studies on the mode of action of the glyophosate tolerant bacterium, the authors came to the conclusion that *P. resinovorans* SZMC 25872 may be inhibiting *A. tumefaciens* SZMC 14557 through extracellular enzyme activities, new bioactive metabolites, or siderophore-mediated suppression.

In the article by Saikia et al., the authors dealt with endophytic Actinobacteria’s phylogenetic association with specific orchid species of Assam, India and their function in promoting the growth and development of plants and inhibiting the growth of phytopathogens. These isolates were classified into six families and eight genera based on the 16S rRNA gene sequence, with *Streptomyces* being the most prevalent. *Streptomyces* was followed by *Actinomadura, Nocardia, Nocardiopsis, Nocardioides, Pseudonocardia, Microbacterium*, and *Mycolicibacterium*. For *in vitro* extracellular enzyme synthesis, PGP, and biocontrol activity against fungal phytopathogens, the majority of the strains showed promising results. The growth of chili plants could be accelerated by two strains. These powerful strains’ ethyl acetate extract underwent GC-MS analysis, which identified 2,4-bis-(1,1-dimethylethyl) as the main metabolite of the antimicrobial component phenol, along with other bioactive compounds that are favorable for plant growth and antifungal properties.

Using taxonomic analytical tools, Fan et al. revealed in their article that the most prevalent genus was *Delftia* and that the major phyla belonged to Proteobacteria and Actinobacteriota. The authors discovered that the endobacterial colonization of *Eichhornia crassipes* underwent systematic changes, with a decline in diversity from root to leaf, suggesting that plant actively participated in endobacteriome selection. Given that *E. crassipes* is a rich source of bacterial strains, many of which show promise as potential bioremediators or bacteria that can promote plant development, it is possible to explore these strains in the development of more climate change-resistant agricultural and environmental practices.

Sinha et al. reported the isolation and characterization of seed microbiomes of eight citrus species and identified nine promising bioinoculants for the development of a microbial consortium with multifaceted PGP traits for better fitness of citrus crops in acidic soil, with special reference to Purvanchal Himalaya, India.

In the article by Yadav et al., the authors highlighted the role of auxins, especially IAA, produced by *Microbacterium testaceum* Y411 in association with the orchid *Rhynchstylis retusa* and showed that they had good potential for improving growth. Additionally, earlier studies showed that auxin is produced by bacterial species from genera such as *Microbacterium*, *Pseudomonas, Stenotrphomonas, Bacillus, and Enterobacter*, which are associated with orchids (Jakubska-Busse et al., 2021) and can improve the growth of these plants (Shah et al.).

The review by Sharma et al. draws attention to a number of biotic stress-causing variables that are responsible for the production of an alternate root exudate composition that affects the rhizosphere microbiota. When root exudates are secreted, they act as a signaling molecule that the rhizosphere bacteria and nearby plants can use to determine the plant’s stress condition. Under biotic stress circumstances, certain exudates are secreted, changing the composition of the rhizospheric microbial community and enhancing plant health by inducing SAR or ISR. Agronomically useful features linked with CWRs’ root exudation may also be used in the future to create crops that are pest-resistant, fertilizer-independent, or weed-suppressive (Preece and Peñuelas). Additionally, investigating the rhizosphere microbiome composition of C3 and C4 plants under various biotic conditions would aid in the development of specialized agronomic approaches for these plant species.

In the review presented by Zulfiqar et al., the authors attempted to summarize the available information on Cd uptake, toxicity, and methods of bioremediation. In this context, the authors showed how soil microorganisms may help in improving Cd-tolerance by synthesizing IAA, which is known to have a role in induced tolerance. An improvement in IAA production has also been shown to induce Pse-w-MT Cd tolerance in *Pisum sativum* L. (Huang et al., 2016). Different PGPRs create extracellular polymeric substances (EPSs), mucopolysaccharides, and proteins that aid in binding harmful HMs for healthy plant growth, mitigating the toxic effects of Cd pollution by reducing Cd uptake.

The research articles and reviews presented in this Research Topic have dealt with various aspects of plant probiotics, emphasizing the beneficial roles of such microorganisms and how they can be put to greater use for sustainable agriculture.

## Author contributions

UC: Writing – original draft, Writing – review & editing. PR: Writing – review & editing. Both authors contributed to the article and approved the submitted version.
